# Transcriptomic and metabolic signatures of neural cells cultured under a physiologic-like environment

**DOI:** 10.1016/j.jbc.2024.107937

**Published:** 2024-10-28

**Authors:** Emilio Fernandez, Moussa Warde, Israel Manjarres-Raza, Veronica Bobo-Jimenez, Maria Martinez-Luna, Carlos Vicente-Gutierrez, Dario Garcia-Rodriguez, Daniel Jimenez-Blasco, Angeles Almeida, Juan P. Bolaños

**Affiliations:** 1 Institute of Functional Biology and Genomics, University of Salamanca, CSIC, Salamanca, Spain; 2 Institute of Biomedical Research of Salamanca, University Hospital of Salamanca, University of Salamanca, CSIC, Salamanca, Spain; 3 Centro de Investigación Biomédica en Red sobre Fragilidad y Envejecimiento Saludable (CIBERFES), Instituto de Salud Carlos III, Madrid, Spain

**Keywords:** astrocyte, neuron, energy metabolism, transcriptomics, glycolysis, hypoxia

## Abstract

Cultured brain cells are used conventionally to investigate fundamental neurobiology and identify therapeutic targets against neural diseases. However, standard culture conditions do not simulate the natural cell microenvironment, thus hampering *in vivo* translational insight. Major weaknesses include atmospheric (21%) O_2_ tension and lack of intercellular communication, the two factors likely impacting metabolism and signaling. Here, we addressed this issue in mouse neurons and astrocytes in primary culture. We found that the signs of cellular and mitochondrial integrity were optimal when these cells were acclimated to grow in coculture, to emulate intercellular coupling, under physiologic (5%) O_2_ tension. Transcriptomic scrutiny, performed to elucidate the adaptive mechanism involved, revealed that the vast majority of differentially expressed transcripts were downregulated in both astrocytes and neurons. Gene ontology evaluation unveiled that the largest group of altered transcripts was glycolysis, which was experimentally validated by metabolic flux analyses. This protocol and database resource for neural cells grown under *in vivo*-like microenvironment may move forward the translation of basic into applied neurobiological research.

The brain is a high energy-demanding tissue that fully relies on O_2_ for its functions. The *in vivo* partial O_2_ tension (pO_2_) in mouse cerebral cortex ranges, according to the distance from arterioles, from 2.6% to 7.8% during normoxia, and it decays to 0.26% to 2.34% during hypoxia (1). Thus, *in vivo* brain cells under physiologic conditions are exposed to pO_2_ values that are far below the atmospheric 21% pO_2_ routinely used in culture (2, 3). On the other hand, astrocytes surround neuronal bodies and synapses, where they sense neuronal activity, fine-tune synaptic transmission (4) and sustains neuronal metabolism (5). In contrast, except works specifically studying cell-cell interactions, neural cells are commonly cultured in isolation thus losing the intercellular communication that grants *in vivo* brain functioning. To circumvent these drawbacks, cells are often grown either at low pO_2_ (6, 7, 8) or seeded in coculture (9, 10) approaches that do not preserve the concerted impact of physiologic pO_2_ and intercellular coupling together. It is therefore plausible to hypothesize that the current knowledge on neuronal and astrocytic metabolic signatures obtained from cultured cells largely differs from *in vivo*. Still, the use of cultured brain cells is a powerful approach to understand fundamental neurobiology and identify molecular targets against diseases. Here, we propose that neurons and astrocytes adapted to grow under physiologic pericellular pO_2_ while preserving intercellular metabolite exchange provide an improved model *in vitro* to recapitulate *in vivo*-like brain microenvironment.

## Results and discussion

**Figure 1:**
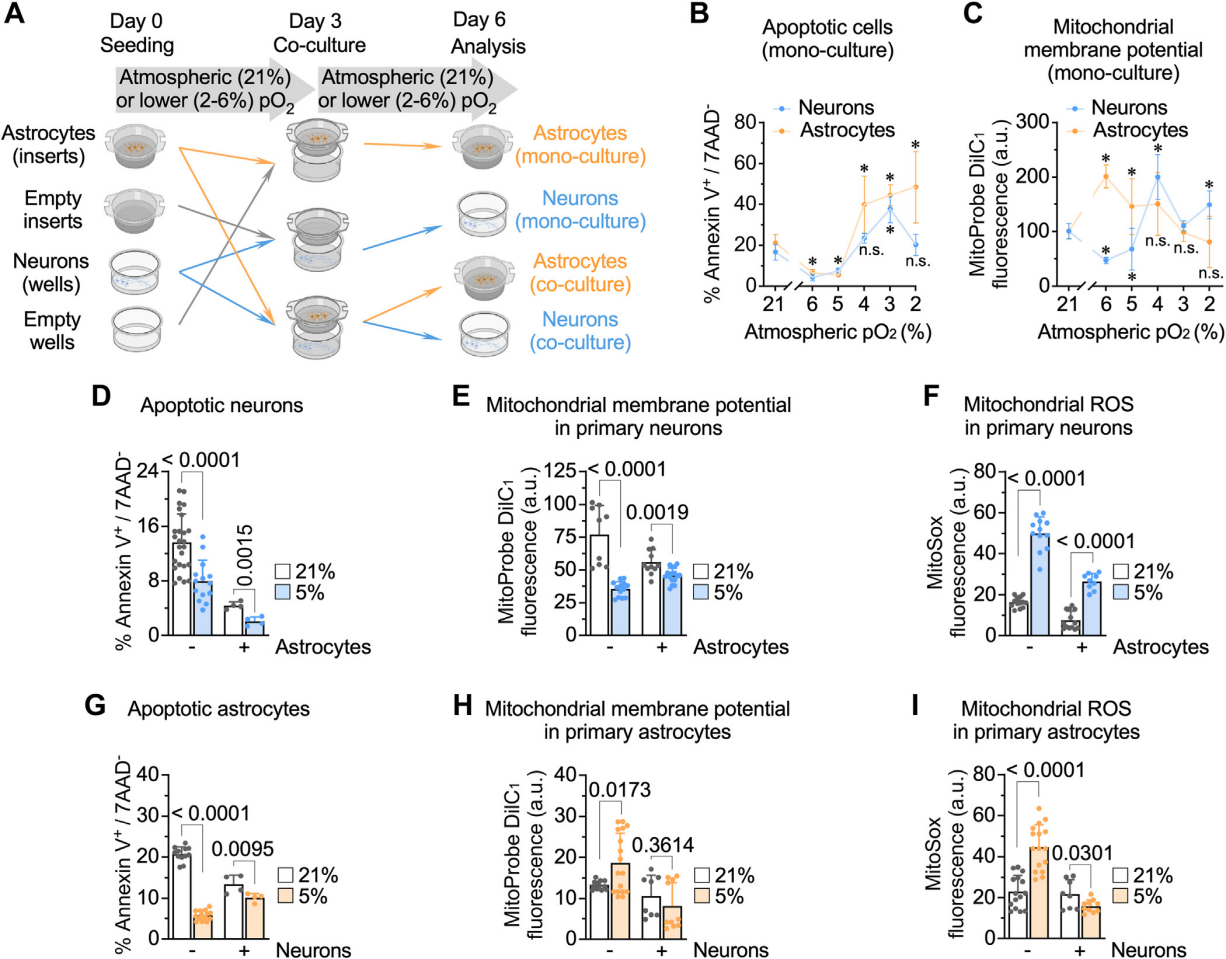
**Incubation of astrocytes and neurons cultured under physiologic-like conditions induce changes in cell viability and mitochondrial fitness.***A*, strategy used to incubate astrocytes and neurons either in monoculture or coculture at different pO_2_. *B* and *C*, apoptotic cell death (*B*) and mitochondrial membrane potential (Δψ_m_) (*C*) in primary astrocytes and neurons grown in monoculture at atmospheric (21%), 6, 5, 4, 3 or 2% pO_2_ for 6 days. Data are mean ± SD. *p* value is indicated; n = 3 to 40 independent culture preparations; two-way ANOVA followed by Tukey. *D*–*F*, apoptotic cell death (*D*), (Δψ_m_ (*E*) and mitochondrial ROS (*F*) in primary neurons grown for 6 days in monoculture, or for 3 days in monoculture plus three further days in coculture with astrocytes, at atmospheric (21%) or 5% pO_2_. Data are mean ± SD. *p* value is indicated; n = 4 to 25 (*D*), n = 9 to 16 (*E*) or n = 9 to 13 (*F*) independent culture preparations; multiunpaired *t* test, two-sided. *G*–*I*, apoptotic cell death (*G*), (Δψ_m_ (*H*) and mitochondrial ROS (i) in primary astrocytes grown in monoculture or coculture with neurons, at atmospheric (21%) or 5% pO_2_. Data are mean ± SD. *p* value is indicated; n = 4 to 14 (*G*), n = 8 to 17 (*H*) or n = 8 to 15 (*I*) independent culture preparations; multiunpaired Student’s *t* test, two-sided. pO_2_, partial oxygen tension; ROS, reactive oxygen species.

To ascertain the impact of pO_2_ at physiologic range on astrocytes and neurons welfare, these cells were grown in monoculture for 6 days at either the atmospheric 21% pO_2_ or at 6, 5, 4, 3, and 2% pO_2_ (Fig. 1*A*). The proportion of apoptotic cells, according to flow cytometry analysis of annexin V^+^/7-amino-actinomycin D (7AAD)^-^ staining, decreased at 6% and 5% pO_2_ (Fig. 1*B*). However, at lower pO_2_ (4%, 3%, and 2%) the proportion of apoptotic cells progressively increased (Fig. 1*B*). The mitochondrial membrane potential (Δψ_m_), an index of mitochondrial integrity, increased in astrocytes at 6 and 5% pO_2_, but decreased at lower pO_2_ (Fig. 1*C*); however, Δψ_m_ in neurons decreased as from 6% pO_2_ (Fig. 1*C*). These results indicate that the reduction of pO_2_ to values of 6 and 5% does not cause monocultured cell damage, although the mitochondrial integrity was better preserved in astrocytes than in neurons. Given that the smallest pO_2_ value at which survival was maximal is 5%, matching the averaged *in vivo* brain pO_2_ in normoxia (1), we selected this pO_2_ value for the following experiments.

To assess if the presence of adjacent astrocytes altered the cellular and mitochondrial welfare of neurons, astrocytes were seeded in cell culture inserts and incubated at 21 or 5% pO_2_ for 3 days; in parallel, neurons were seeded in the cell plates and incubated under the same conditions (Fig. 1*A*). At day 3, inserts containing astrocytes were placed on the neuronal layer and further coincubated at either 21 or 5% pO_2_ for three more days (Fig. 1*A*). As controls, we either used empty inserts on neurons or astrocytes-containing inserts on empty plates (Fig. 1*A*). Under these conditions, we observed that the presence of astrocytes significantly improved the viability of neurons at 21% and 5% pO_2_, being these values minimum at 5% (Fig. 1*D*). This result is in good agreement with previously reported data in human cancer cell lines indicating improved viability when incubated at low pO_2_ (7). Furthermore, although astrocytes induced a reduction in neuronal Δψ_m_ both at 21 and at 5% pO_2_ (Fig. 1*E*), Δψ_m_ values at 5% pO_2_ were significantly higher in cocultured neurons when compared with isolated neurons (Fig. 1*E*). These data indicate that the presence of neighboring astrocytes improves neuronal survival and counteracts the mitochondrial Δψ_m_ loss observed in neurons incubated alone at 5% pO_2_.

To ascertain the impact of pO_2_ and coculture on the levels of redox stress in these cells, we next measured mitochondrial reactive oxygen species (mROS). Reduction of pO_2_ from 21 to 5% increased mROS in neurons (Fig. 1*F*). However, the presence of astrocytes strongly attenuated this effect (Fig. 1*F*). On the other hand, the presence of contiguous neurons improved the survival of astrocytes at 21% pO_2_ and slightly increased the apoptotic death at 5% pO_2_ to reach the values found at 21% pO_2_ (Fig. 1*G*). Likewise, the increased Δψ_m_ observed in astrocytes when switched from 21 to 5% pO_2_ was counteracted by adjacent neurons (Fig. 1*H*), indicating that neurons do not alter astrocytic mitochondrial integrity in the switch 21 to 5% pO_2_. Finally, reduction of pO_2_ from 21 to 5% increased mROS in astrocytes, an effect that was strongly attenuated by the presence of neurons (Fig. 1*I*). Altogether, these data illustrate that the close proximity of neurons and astrocytes adapted to grow in coculture at normoxic 5% pO_2_ improves the health hallmarks of the isolated cells cultured at atmospheric 21% pO_2_.

**Figure 2:**
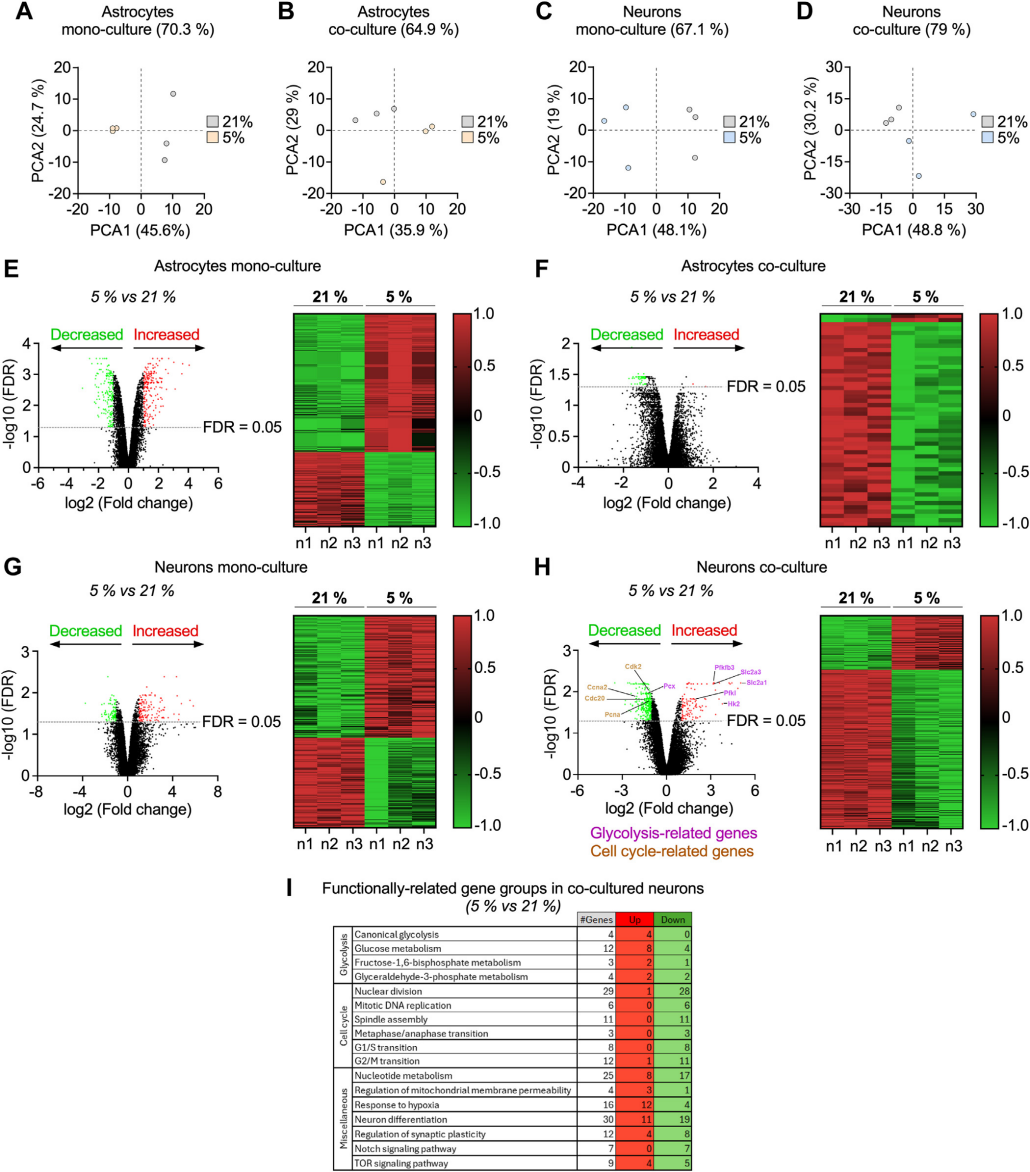
**Transcriptomic analysis in astrocytes and neurons cultured under physiologic-like conditions suggests glucose energy metabolism and differentiation pathways reprogramming.***A*–*D*, PCA analysis of the transcriptome of astrocytes grown for 6 days in monoculture (*A*), or for 3 days in monoculture plus three further days in coculture with neurons (*B*); and neurons grown for 6 days in monoculture (*C*), or for 3 days in monoculture plus three further days in coculture with astrocytes (*D*), at atmospheric (21%) or 5% pO_2_. *E*–*H*, transcripts altered in cells grown at 5% *versus* at 21% pO_2_ in mono- (*E*, *G*) or coculture (*F*, *H*). Data are mean log_2_ expression levels and -log_10_ FDR values from n = 3 independent culture preparation for each condition; Empirical Bayes method followed by Benjamini-Hochberg correction. *I*, functional annotations of the transcripts most significantly altered in neurons grown at 5% *versus* at 21% pO_2_ in coculture with astrocytes. Fisher’s Exact test followed by Benjamini-Hochberg correction. FDR, false discovery rate; PCA, perchloric acid; pO_2_, partial oxygen tension.

Next, we aimed to identify the potential mechanism(s) accounting for the adaptation of neurons and astrocytes to these physiologic-like conditions. To do so, we performed transcriptomics, and analysis of the transcripts of these cells cultured alone or in coculture at 5% or 21% pO_2_ revealed differentiated groups according to the perchloric acid analysis (Fig. 2, *A–D*). A slight majority of differentially expressed (−2< fold change >2) transcripts were upregulated in astrocytes (347 upregulated genes, 65.1%; false discovery rate (FDR) < 0.05) (Fig. 2*E*) and in neurons (143 upregulated genes, 57.7%; FDR < 0.05) (Fig. 2*G*) when switched from 21 to 5% pO_2_ (see also the Source Data file). This result contrasts with those (6) previously reported data indicating an even up and downregulated proportion of genes in astrocytes grown at 4% pO_2_. However, we further show that the vast majority of transcripts were downregulated in the cocultured cells at 5% *versus* 21% pO_2_ both in astrocytes (48 downregulated genes, 96%; FDR<0.05) (Fig. 2*F*) and in neurons (322 downregulated genes, 74.5%; FDR<0.05) (Fig. 2*H*). Functional annotation by biological processes of the differentially expressed transcripts in cocultured neurons in the switch 21 to 5% pO_2_ evocates increased glycolysis and reduced cell-cycle related pathways (Fig. 2*I*).

**Figure 3:**
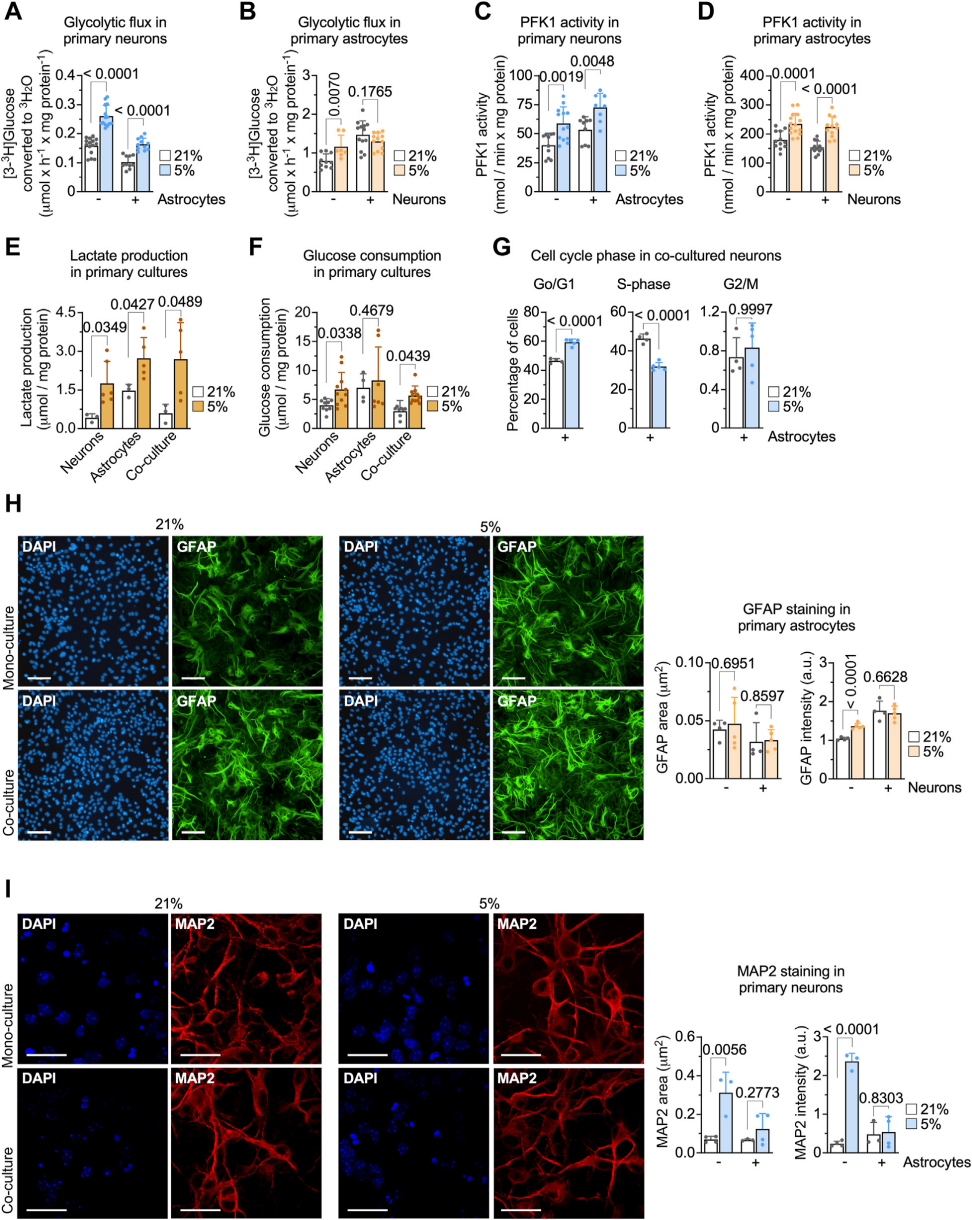
**Functional analysis reveals glycolysis and differentiation adaptations in astrocytes and neurons cultured under physiologic-like conditions.***A* and *B*, rates of glycolytic flux in neurons (*A*) or astrocytes (*B*) grown in monoculture for 6 days, or in monoculture for 3 days plus three further days in coculture, at 21 or 5% pO_2_. Data are mean ± SD. *p* value is indicated; n = 10 to 15 (*A*), n = 7 to 13 (*B*) independent culture preparations; multiunpaired *t* test, two-sided. *C* and *D*, PFK1 activities in neurons (*C*) or astrocytes (*D*) grown in monoculture or coculture at 21 or 5% pO_2_. Data are mean ± SD. *p* value is indicated; n = 8 to 13 (*C*), n = 12 to 15 (*D*) independent culture preparations; multiunpaired *t* test, two-sided. *E*, lactate production in neurons or astrocytes grown in monoculture or coculture at 21 or 5% pO_2_. Data are mean ± SD. *p* value is indicated; n = 3 to 6 independent culture preparations; multiunpaired *t* test, two-sided. *F*, glucose consumption in neurons or astrocytes grown in monoculture or coculture at 21 or 5% pO_2_. Data are mean ± SD. *p* value is indicated; n = 4 to 11 independent culture preparations; multiunpaired *t* test, two-sided. *G*, cell cycle analysis in neurons cocultured with astrocytes at 21 or 5% pO_2_ for 3 days. Data are mean ± SD. *p* value is indicated; n = 4 to 5 independent culture preparations; multiunpaired *t* test, two-sided. *H*, GFAP staining in astrocytes grown in monoculture or coculture with neurons at 21 or 5% pO_2_. Data are mean ± SD. *p* value is indicated; n = 4 to 5 independent culture preparations; multiunpaired *t* test, two-sided. *I*, Tuj1 staining in neurons grown in monoculture or coculture with astrocytes at 21 or 5% pO_2_. Data are mean ± SD. *p* value is indicated; n = 3 to 4 independent culture preparations; multiunpaired *t* test, two-sided. The scale bar represents 25 μm. GFAP, glial fibrillary acidic protein; PFK1, 6-phosphofructo-1-kinase; pO_2_, partial oxygen tension.

**Figure S1:**
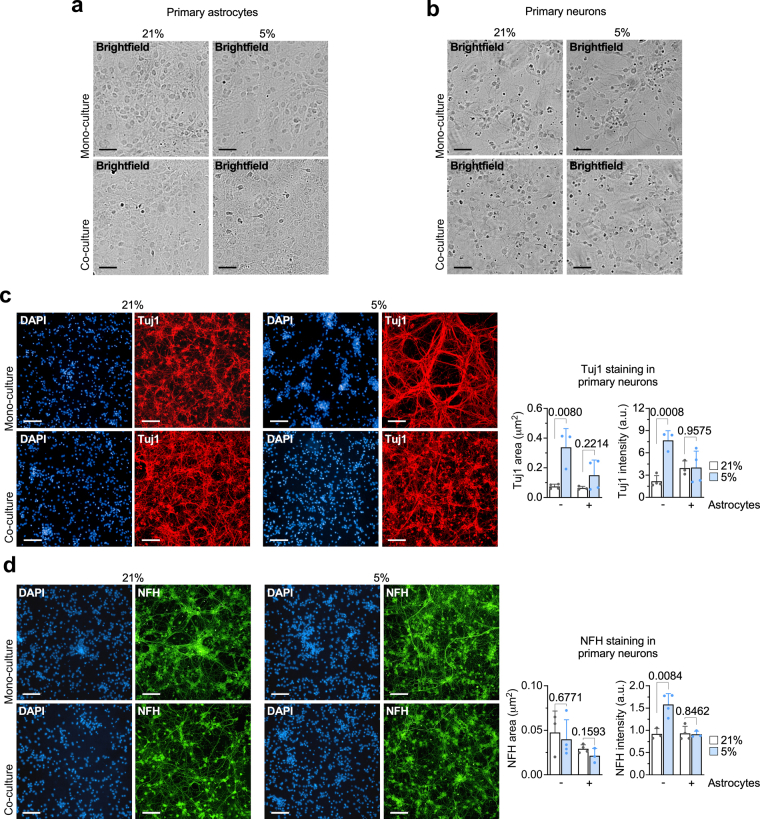


Given the central role of glycolysis in cell metabolism, we functionally confirmed the observed alterations in the glycolytic transcripts by analyzing the glycolytic flux using [3-^3^H]glucose (11). We observed that glycolytic rate at 21% pO_2_ in neurons alone was lower than that in astrocytes (Fig. 3, *A* and *B*), in agreement with previous data (11). Moreover, switching to 5% pO_2_ promoted an increase in glycolysis in both cell types (Fig. 3, *A* and *B*). Interestingly, neuronal glycolysis at 21% pO_2_ decreased by the presence of astrocytes (Fig. 3*A*), an effect that was counteracted by switching pO_2_ to 5% (Fig. 3*A*). In contrast, the presence of neurons increased the rate of glycolysis in astrocytes, both at 21% and 5% pO_2_ (Fig. 3*B*). In sum, astrocytes attenuates neuronal glycolysis, and neurons stimulates astrocytic glycolysis to reach values that, at the physiologic 5% pO_2_, the rate of astrocytic glycolysis, is > 2-fold higher than in neurons (Fig. 3, *A* and *B*). These changes paralleled those of the transcripts abundances of the glycolysis rate-limiting enzyme 6-phosphofructo-1-kinase (PFK1) (Fig. 2, *F* and *H*; see, also, the Source Data), the enzymatic activity of which was functional confirmed (Fig. 3, *C* and *D*). Furthermore, the alterations observed in PFK1 activity and glycolytic fluxes were also confirmed by those in lactate release and glucose consumption (Fig. 3, *E* and *F*). The increased proportion of transcripts evocating cell cycle differentiation in neurons cocultured with astrocytes in the switch 21 to 5% pO_2_ (Fig. 2*I*) were confirmed both by flow cytometric analysis of the cell cycle phases (Fig. 3*G*) and by immunostaining (Figs. 3, *H* and *I*; S1).

In conclusion, we show that neurons and astrocytes adapted to grow in culture at physiologic pO_2_ while preserving intercellular metabolite exchange mimics features of the *in vivo*-like physiologic conditions. Under these conditions, our findings confirm the previously reported hyperglycolytic phenotype of astrocytes when compared with that of neurons (5, 11, 12) and further show that, when these cells are cultured under a physiologic-like environment that preserves the intercellular cooperation, this metabolic peculiarity is emphasized along with neuronal differentiation. Moreover, our data also reveal that, besides an atmosphere resembling *in vivo* pO_2_ (8, 13, 14), the intercellular coupling is a condition needed to adequately recreate the brain physiologic microenvironment when investigating neural cells in culture. The herein developed protocol and reported database resource of neural cells grown under *in vivo*-like microenvironment may help researchers to translate basic into applied neurobiological research.

## Experimental procedures

### Animals

C57BL/6J mice were supplied by the Animal Experimentation Facility of the University of Salamanca. Breeding was in cages with a 12 h light-dark cycle, 45 to 60% of humidity, and 20-25 °C of room temperature. Animals were fed *ad libitum* with a standard solid diet (17% proteins, 3% lipids, 58.7% carbohydrates component, 4.3% cellulose, 5% minerals, and 12% humidity) and with free access to water. All animal handlings and procedures were in agreement with the current regulation from the European commission (2014/11/EU) and Spanish legislation (Law 6/2013) related to accommodation and experimental animals’ care. All the protocols were approved by Bioethics Committee of the University of Salamanca.

### Primary cell cultures and cocultures

Cortical neurons in primary culture were prepared from E15 days murine fetuses (15) and seeded at 2.0 × 10^5^ cells/cm^2^ in different size plastic culture plates (Nunc and Costar; Corning for coculture), previously coated with poly-D-lysine (10 μg/ml; Sigma-Aldrich) in Neurobasal-A (Thermo Fisher Scientific) supplemented with 5.5 mM glucose, 2 mM glutamine, and 2% B-27 MAO) (Life Technologies). Cultures were incubated at 37 °C either in a humidified 5% CO_2_-containing atmosphere (21% of O_2_), or at lower O_2_ concentrations (2–6%) in an oxygen regulated workstation (Baker-Ruskinn InVivo400), able to maintain specific and stable low oxygen concentrations for long periods of time. When the pO_2_ was lower than the atmospheric pO_2_, the medium was equilibrated at the hypoxia incubator chamber pO_2_ before use. To do this, the medium was stirred in an open flask inside the hypoxia incubator while the medium pO_2_ was monitored using a Crison OXI45 oximeter (Hach). The time required to stabilize the medium at the required pO_2_ was <10 min (not shown). Medium was renewed on a 3-day basis, and cells used at 6 days. Astrocytes in primary culture were obtained from neonates from 0 to 24 h of age (15). Cellular suspension was seeded at 2.5 × 10^5^ cells/cm^2^ in 175 cm^2^ culture flasks (BD, Falcon) in low glucose (5.5 mM) Dulbecco’s modified Eagle’s medium supplemented with 10% fetal calf serum (FCS). Medium was changed twice per week. At day 7, flasks were shaken at 180 r.p.m for 16 h in an orbital shaker, under the same culture conditions. Medium with floating cells was discarded and the astrocyte-enriched cells monolayer was harvested by trypsinization for 4 min. The reaction was stopped by adding FCS at a final concentration of 10% and cell suspension was centrifuged at 500*g* for 5 min. Cells were resuspended in 10 ml of Neurobasal-A supplemented with 5.5 mM glucose, 2 mM glutamine, and 2% B-27, then seeded at 1 × 10^5^ cells/cm^2^ in different size plastic plates and incubated at 37 °C either in a humidified 5% CO_2_-containing atmosphere (21% of O_2_), or at lower O_2_ concentrations (2–6%). Medium was replaced every 3 days and cell used after six additional days in culture. To obtain astrocyte-neuronal cocultures, astrocytes at 8 days *in vitro* were reseeded on semipermeable polyester Transwell membrane inserts (0.4 μm pore size, Corning) and incubated at either 21 or 2 to 6% pO_2_. At day 11 *in vitro*, astrocyte-containing inserts were placed over 3 days *in vitro* neurons and cocultured in Neurobasal-A supplemented with 2 mM glutamine, 5.5 mM glucose, 0.22 mM pyruvate, and 2% B27 MAO supplement for three more days at either 21 or 2 to 6% pO_2_.

### Analysis of apoptotic cell death

Apoptosis was assessed by Annexin V/7-AAD Apoptosis Detection Kit by flow cytometry. After 15 min of incubation at 37 °C with annexin V and 7-AAD in binding buffer (100 mM Hepes, 140 mM NaCl, and 2.5 mM CaCl_2_), cells were carefully detached from the plates using 1 mM EDTA (tetrasodium salt) in PBS (pH 7.4) for neurons or trypsin/EDTA followed by 10% FCS for astrocytes, centrifuged for 5 min at 500*g* and then resuspended in PBS (136 mM NaCl, 2.7 mM KCl, 7.8 mM Na_2_HPO_4_ 2H_2_O, and 1.7 mM KH_2_PO_4_ pH 7.4), prior to quantification on a FACScalibur flow cytometer (15 mW argon ion laser tuned at 488 nm) and analyzed using CellQuest PRO (https://www.bdbiosciences.com/content/dam/bdb/marketing-documents/14_cellquest_prosoft_acquisit.pdf) and Paint-A-Gate PRO (BD Biosciences; https://www.bdbiosciences.com/en-us/products/instruments/software-informatics/instrument-software/bd-paint-a-gate-pro-for-windows.649728) software. Only annexin V-stained cells that were 7-AAD-negative were considered apoptotic.

### Mitochondrial membrane potential

Δψ_m_ was assessed by flow cytometry using the probe DiIC1(5) (1,1′,3,3,3′,3′-hexamethylindodicarbo-cyanine iodide) (Life Technologies) (50 nM), a cationic cyanine dye that accumulates in the mitochondrial matrix according to the inner mitochondrial membrane potential. Cells were smoothly harvested, centrifuged at the appropriate pO_2_, and suspended in PBS. The mitochondrial uncoupler carbonyl cyanide 3-chlorophenylhydrazone (10 μM) was used to define the full depolarized value.

### Mitochondrial reactive oxygen species

Cells were incubated with 2 μM MitoSox-Red (Invitrogen, Thermo Fisher Scientific) in Hank’s balanced salt solution (134.2 mM NaCl; 5.26 mM KCl; 0.43 mM KH_2_PO_4_; 4.09 mM NaHCO_3_; 0.33 mM Na_2_HPO_4_·2H_2_O; 5.44 mM glucose; 20 mM Hepes [4-(2-hydroxyethyl)-1-piperazineethanesulfonic acid]; 4 mM CaCl_2_·2H_2_O; pH 7.4) for 30 min, washed with PBS and carefully detached from the plate using trypsin/EDTA. As a positive control, 10 μM antimycin A (15 min). MitoSox-Red fluorescence was assessed by flow cytometry.

### Determination of PFK1 activity

Cells were harvested, pelleted, and resuspended in 100 μl of PFK1 storage buffer [20 mM KHPO4, 0.1 mM EDTA, 20% glycerol, 10 mM DTT, 10% fructose-6-phosphate, 2% phenylmethylsulfonyl fluoride], at pH 7.4. Cells were disrupted by freeze/thawing and used for PFK1 activity determination spectrophotometrically by assessing the decrease in NADH(H^+^) absorbance at 340 nm in buffer containing imidazole (50 mM), MgCl_2_ (1 mM), NADH(H^+^) (0.1 mM), ATP (1 mM), fructose-6-phosphate (2 mM), fructose-2,6-bisphosphate (0.046 mM), aldolase (0.5 U/ml), triose phosphate isomerase (10.29 U/ml) and glycerol-3-phosphate dehydrogenase (0.97 U/ml) at 0H 7.4. The determinations were carried out against a blank lacking the PFK1 substrate, fructose-6-phosphate (12).

### Glycolytic flux

This was performed by determining ^3^H_2_O production from D-[3-^3^H]glucose, as previously described (11), with some modifications. Briefly, culture medium was discarded, and cells were washed with PBS. When cells were grown in coculture, the inserts were separated from the plates and both incubated with 1.5 ml of Neurobasal-A-minus antioxidants (MAO) medium, supplemented with 5.5 mM D glucose and 5 μCi of D-[3-^3^H]glucose per sample, for 2 h at the appropriate pO_2_. After this incubation, the media were transferred to glass Erlenmeyer flasks with a central well harboring an Eppendorf tube containing 1 mL of water. The flasks were sealed with rubber caps and 0.2 ml of 20% w/v perchloric acid (ClO_4_H) was injected through the cap and incubated for 96 h in a thermostatic orbital shaker at 36 °C to allow equilibration of ^3^H_2_O between the Eppendorf tube and the main well. The radioactivity in the Eppendorf tubes was measured by liquid scintillation counting to assess the glycolytic flux. The efficiency of ^3^H_2_O (28%) was taken into account for the calculation. A triplicate was prepared for each biological replica. The results were expressed as nmol of D-[3-^3^H] glucose converted into ^3^H_2_O per h and per mg protein.

### Lactate and glucose determinations

Lactate concentrations were measured in 100 μl of deproteinized culture medium samples taken 2 h after medium change, spectrophotometrically as described (11).

### Determination of incorporated bromodeoxyuridine

Neurons were fixed, permeabilized, and resuspended with DNase (300 μg/ml) during 1 h at 37 °C. After staining with allophycocyanin fluorochrome-conjugated monoclonal anti-bromodeoxyuridine antibody, the fluorescent signals were analyzed by flow cytometry.

### RNA isolation

Cultures of 1.5 to 2 × 10^6^ cells were washed twice with PBS and lysed with 200 μl/well of RNeasy lysis buffer (Qiagen). Cellular lysates were centrifuged for 3 min at 13,000*g*, and the supernatants were collected. One volume of 70% ethanol was added to the supernatants and 700 μl of the sample were transferred to the RNeasy Mini spin column. Samples were centrifuged for 15 s at ≥ 8000*g* and the flow-through was discarded. DNase digestion was performed by adding 80 μl/sample of DNase I mix (10 μl DNase I + 70 μl DNase buffer 10x) for 15 min. DNase reactions were stopped by adding 700 μl of Buffer RW1 (Quiagen) and columns containing RNA samples were washed with two cycles of RPE buffer. RNA samples were collected with 50 μl of RNase-free water followed by 1 min centrifugation at ≥ 8000*g*. The RNA concentrations and purity were verified by Nanodrop 2000 (Thermo Fisher Scientific) and kept at −80 °C until their use.

### Microarrays processing and analysis

RNA samples of 100 ng/μl were used for Exon arrays and Gene arrays. Agilent Bioanalyzer was used to check quality and purity of the samples previous to Affymetrix microarray hybridization using Clariom D assays, mouse tools. Data were analyzed using the Transcriptomic Analysis Console (Thermo Fisher Scientific; https://www.thermofisher.com/es/es/home/life-science/microarray-analysis/microarray-analysis-instruments-software-services/microarray-analysis-software/affymetrix-transcriptome-analysis-console-software.html). Sample data were normalized forming subgroups prior to the analysis. Transcripts were filtered, and transcripts aligned in noncoding regions were discarded. A threshold of −2 < fold change > 2 and FDR < 0.05 was established to consider a gene as a significant difference within each comparison. Pathway analysis for cocultured neurons was performed running a statistical overrepresentation test in the PantherDB online tool. Pathways with an FDR < 0.05 were considered to be significantly enriched.

### Immunocytochemistry

Neurons and astrocytes grown on glass coverslips were fixed with formalin solution, neutral buffered, 10% histological tissue fixative (MKCS39994; Sigma-Aldrich) for 1 h and immunostained with mouse anti-MAP2 (ab11267; Abcam; 1:500), rabbit anti-beta III tubulin (Tuj1, ab18207; Abcam; 1:1000) or rabbit anti-Neurofilament H (NFH, AB1989; Millipore; 1:200) for neurons, and rabbit anti-GFAP (G9269; Sigma-Aldrich; 1:200) for astrocytes. Immunolabeling was detected using anti-mouse IgG-Cy2, anti-rabbit IgG-Cy2 or anti-rabbit IgG-Cy3 (1:500; Jackson ImmunoResearch). Coverslips were washed with PBS and mounted in SlowFade light antifade reagent (Invitrogen) or glass slides, confocal, and brightfield images were acquired using Operetta microscope with a 40X Air, 0.6 NA, and 20X Air, 0.4NA objectives (PerkinElmer). Images were analyzed using building blocks from Harmony software (PerkinElmer; https://www.chumontreal.qc.ca/sites/default/files/2023-05/PDF1.pdf).

### Statistical analysis

For simple comparisons, we used unpaired two-tailed Student’s *t* test. For other multiple-values comparisons, we used two-way ANOVA followed by Tukey or Bonferroni *post hoc* tests. For the microarray analysis, we used the Empirical Bayes method followed by Benjamini-Hochberg correction for multiple comparisons. For the pathway analysis, we used the online tool PantherDB v18.0, performing a statistical overrepresentation test with the differentially expressed genes applying the Fisher’s Exact test followed by Benjamini-Hochberg correction. All tests used are indicated in each figure legend. The statistical analysis was performed using the GraphPad Prism v8 software (https://www.graphpad.com/features). The number of biologically independent culture preparations used per experiment are indicated in the figure legends. Data are presented as the mean ± standard deviation (SD).

## Data availability

Source Data for all figures are provided along this manuscript.

## Supporting information

This article contains Supporting Information.

## Conflicts of interests

The authors declare that they have no conflicts of interest with the contents of this article.

## Supporting information

Source Data Figure S1

Statistics Table
